# The Effect of Systemic Arterial-Line Leukocyte Filtration on the Outcome of Adult Patients Undergoing Cardiac Surgery

**DOI:** 10.5812/ircmj.10912

**Published:** 2013-05-05

**Authors:** Hamidreza Taghipour, Hamid Shafiei, Omid Assar, Mohammad Saaid Ghiasi

**Affiliations:** 1 Trauma research center, Baqiyatallah University of Medical Sciences, Tehran, IR Iran; 2 Cardiac Surgery Ward, Baqiyatallah Hospital, Tehran, IR Iran; 3 Cardiovascular Research Center, Baqiyatallah University of Medical Sciences, Tehran, IR Iran; 4 Jamaran Heart Hospitals, Tehran, IR Iran

**Keywords:** Cardiopulmonary Bypass, Leukocytes, Thoracic Surgery

## Abstract

**Background:**

It is known that cardiopulmonary bypass causes an inflammatory reaction with associated morbidity and mortality. Several anti-inflammatory strategies have been implemented to reduce this response, including leukocyte removal from the circulation using specialized filters.

**Objectives:**

The aim of this randomized clinical study was to assess the impact of arterial-line systemic leukocyte filtration on the postoperative outcome of adult patients undergoing elective cardiac surgery.

**Patients and Methods:**

114 patients undergoing CABG or valve replacement in Baqiyatallah hospital, Tehran, Iran from May to August 2011 were randomly assigned to two groups: with and without leukocyte filtration and their outcomes were compared.

**Results:**

The postoperative intubation time was significantly shorter in patients with leukocyte filters (0.014). There was no significant difference between two groups regarding other outcome relatedvariables.

**Conclusions:**

Systemic arterial leukocyte filtration reduces the intubation time but has no other beneficial effect on the outcome of patients undergoing CABG or valve surgery.

## 1. Background

Cardiopulmonary bypass (CPB) which is widely used in cardiac surgery is associated with an inflammatory response. This inflammatory reaction contributes to an organ dysfunction which may sometimes lead to severe complications or even mortality. It is well-known that leukocytes play a major role in the production of this inflammatory process. So, it seems that removal of leukocytes from the circulation is a reasonable method to reduce tissue injury during CPB. Since the introduction of the first specific leukocyte-depleting filter in 1991, several studies have investigated its efficacy in cardiac surgery with inconclusive results. This may be due to the various strategies of leukocyte depletion regarding the timing and duration of filtration and also focusing on different end points (1).

## 2. Objectives

The aim of this randomized clinical study was to assess the impact of arterial-line systemic leukocyte filtration on postoperative outcome (including respiratory function, myocardial protection, transfusion requirements) of adult patients undergoing elective cardiac surgery.

## 3. Patients and Methods

After institutional ethics committee approval (IRB code:380-date:2011/3/14) and patient informed consent considering national research ethics check list, 114 patients undergoing cardiopulmonary bypass for CABG or valve replacement were randomly assigned to two groups .Random allocation was done using random number generator. 57 patients had an arterial leukocyte filter (LeukoGuard, Pall medical, NY, USA) and for the remaining 57 patients leukocyte filter was not used. The exclusion criteria were: age > 80 years, LVEF < 35%, treatment with steroids or other anti-inflammatory drugs, white blood cell count < 5000/ml, emergency or redo surgery (114 of 138 patients operated during the study period met the inclusion criteria). Premedication, induction and maintenance of anesthesia were the same in all patients. The assembly and conduct of the CPB was also identical in both groups except for the use of leukocyte filter. Most operations were performed during moderate (34 ^o^C) hypothermia. Cold antegrade blood cardioplegia was used and repeated every 20 minutes. The leukocyte filter was connected to the main arterial line and in the circuit the total time of CPB meaning its bypass line was clamped. The ICU personnel were blinded to the use of leukocyte filtration. The patients were extubated when they were fully awake and stable and had satisfactory arterial blood gas levels. Complete blood count, Troponin I and Creatine Phosphokinase-MB (CPK-MB) were measured in all patients preoperatively, upon arrival in the ICU and on the first postoperative day. Arterial blood gases were recorded at 2 hours, 12 hours and 24 hours postoperatively. Transfusion trigger points were Hct = 21 during CPB and Hct = 24 after it. The number of transfused packed cell units for each patient was recorded. Comparisons were accomplished by using T-test (normally distributed data) and Mann-Whitney test (non-normally distributed data) for continuous variables, and chi 2 and Fisher exact test for categorical variables. A value of P < 0.05 was regarded as statistically significant. Data was collected and analyzed by SPSS Ver. 16 software.

## 4. Results

There were no differences in the preoperative data and intraoperative variables except for more prevalence of hypertension in the leukodepleted group](P = 0.01) (Table 1)[. Postoperative leukocyte count was lower in the leukocyte filtered group (P = 0.02). The mean length of intubation was less in the leukodepleted group (P = 0.014) but arterial or mixed venous Po ^2 ^at 2, 12 and 24h were not different in two groups. Cardiac enzymes (Troponin I and CPK-MB) levels in blood at ICU arrival (P = 0.96 and P = 0.87) and first postoperative day (P = 0.91 and P = 0.99) showed no significant differences. The amount of transfused blood was not significantly different in two groups (P = 0.4) (Table 2).

**Table 1.** Detailed Demographic, Clinical Variables and Risk Factors in Two Groups

Demographic Information	Non Leukocyte Filter	Leukocyte Filter	P value
**Gender, No. (%)**			
Male	34(59.6)	41(71.9)	0.167
**Age, y, Mean ± SD**	60.64 ± 10.25	58.58 ± 15.14	0.815
**Ejection Fraction, Mean ± SD**	44.63 ± 8.91	44.16 ± 8.05	0.520
**Weight, kg, Mean ± SD**	75.68 ± 22.13	71.33 ± 11.52	0.454
**Height, cm, Mean ± SD **	161.15 ± 19.74	164.78 ± 8.71	0.708
**Diabetes Mellitus, No. (%) **	32(56.1)	22(38.6)	0.601
**Hypertension, No. (%)**	37(64.9)	24(42.1)	0.015
**COPD, No. (%)**	4(7)	7(12.3)	0.341
**Renal failure, No. (%)**	2(3.5)	2(3.5)	0.999
**Emergency surgery, ** **No. (%) **	4(7)	4(7)	0.999
**History of CVA, No. (%)**	1(1.8)	3(5.3)	0.618
**CPB time, min, Mean ± SD**	59.41 ± 22.96	63.77 ± 24.38	0.178
**Cross clamp time, min, Mean ± SD**	36.89 ± 21.26	41.73 ± 25.66	0.187
**CABG, No. (%) **	50(87.7)	47(82.5)	0.486

**Table 2.** Postoperative Data

Data	Non Leukocyte Filter, Mean ± SD	Leukocyte Filter, Mean ± SD	P value
**Intubation time, h**	8.32 ± 3.86	6.66 ± 2.30	0.014
**Transfused blood (unit)**	1.22 ± 1.32	0.98 ± 1.06	0.409
**WBC (x 103/µL)**	5.89 ± 3.28	4.27 ± 2.82	0.020
**Troponin-I (0) (** **ng** **/ml)**	6.76 ± 17.41	4.30 ± 2.22	0.963
**Troponin-I (24) ,** **ng** **/ml**	3.40 ± 12.91	2.22 ± 8.45	0.918
**CPK (0), ** **ng** **/ml**	16.35 ± 24.79	16.77 ± 21.55	0.877
**CPK(24), ** **ng** **/ml**	18.69 ± 24.68	18.44 ± 20.92	0.656
**PO2 (2), mm Hg**	90.35 ± 79.99	105.66 ± 83.13	0.292
**PO2(12), mm Hg**	145.61 ± 65.40	143.91 ± 72.51	0.998
**PO2 (24), mm Hg**	156.35 ± 64.96	152.32 ± 77.61	0.991

## 5. Discussion

The concept of leukocyte depletion with a leukocyte removal filter was introduced in 1926 by Fleming and Wright (2, 3). In the early 90s, favorable results of animal experiments led to clinical studies and since then various strategies using leukocyte filters in cardiac surgery patients have been explored, Strategy and efficacy of leukocyte depletion. As far as the duration of leukocyte depletion is concerned, there is no consensus so far as to how long it should be. The strategies applied vary regarding the filtration timing (during cross-clamp vs. during reperfusion, intermittent vs. continuous) and the filter position (arterial vs. venous, cardioplegic line, residual blood or a combination of these methods). De Vries, et al. contrasted arterial-line systemic depletion and venous-line systemic depletion during the rewarming phase of CPB and leukofiltration of residual blood during autologous transfusion and found no clinical difference between groups (4). As with the arterial-line filtration starting at the beginning of CPB, the leukocyte filter may catch activated leukocytes by the ”first pass” effect of blood interaction with the foreign material in the CPB circuit (5). This obvious advantage of arterial-line leukocyte filter led us to use this method in our study. However, as the arterial-line filter is employed during the entire period of perfusion, saturation of the filter is possible over the first 30-40 minutes and there is probably little effective capacity of the `exhausted` filter left for depleting leukocytes during the important reperfusion phase and even the trapped leukocytes may act as a potential source of enzyme release and enhance the inflammatory process (5, 6). This fact is ignored in most studies including our own and can bias the results towards no difference between groups. In our study, although postoperative leukocyte count was lower in the leukodepleted group, supporting the efficacy of the leukocyte filter, leukocyte activity was not assessed.

### 5.1. Respiratory Function

Palanzo, et al. reported better arterial Po^2^ at cessation of CPB and significantly shorter ventilation time for those patients undergoing leukodepletion (7). Patel, et al. also reported a significantly shorter time to extubation and improved respiratory quotient (8). Chen, et al. reported a significantly better oxygenation index at 10h in filtered patients, but this was not mirrored in a shorter intubation period (8). In our study, we observed a shorter intubation time in leukocyte filtered patients but Po^2^ was not significantly different in two groups. Time of extubation dependents on many factors and should not be regarded as a proof of better postoperative lung function. This may justify different results observed in clinical studies. Besides, use of more accurate parameters like oxygenation index instead of Po^2^ in our study would compare the groups better.

### 5.2. Myocardial Protection

Most studies report a trend towards a decrease in cardiac enzymes (CPK-MB and Troponin I or T) for systemic filtered patients without, however, recording statistically significant differences (8). In a large randomized trial, there was no difference between the filtered and control group, for either cardiac markers (9). We did not observe any significant difference in postoperative Troponin I level between our study groups, either.

### 5.3. Blood Transfusion

According to the 2011 STS and SCA update of blood conservation clinical practice guidelines, available leukocyte filters placed on the CPB circuit for leukocyte depletion are not indicated for perioperative blood conservation and may prove harmful by activating leukocytes during CPB (10). In our study, the transfusion requirement was not significantly different in two groups. In most studies like our own, leukofiltration do not appear to impact on wound infection or postoperative sepsis. In a study by Tang, et al. although daily fluid balance, serum creatinin and blood urea remained comparable in both groups, sensitive indicators (eg. Urinary microalbumin indexed to creatinin) revealed significantly more truama to both renal tubules and glomeruli system after CPB without leukodepletion (11). Our study also showed no difference between two groups regarding routine renal function tests. Systemic arterial leukocyte filtration reduces the intubation time but has no other beneficial effect on the outcome of patients undergoing CABG or valve surgery.
